# Inhibition of non-small cell lung cancer (NSCLC) growth by a novel small molecular inhibitor of EGFR

**DOI:** 10.18632/oncotarget.3155

**Published:** 2015-02-05

**Authors:** Jinsong Li, Huayun Deng, Meichun Hu, Yuanzhang Fang, Amanda Vaughn, Xiaopan Cai, Leqin Xu, Wei Wan, Zhenxi Li, Shijie Chen, Xinghai Yang, Song Wu, Jianru Xiao

**Affiliations:** ^1^ Department of Orthopedic Oncology, Changzheng Hospital, The Second Military Medical University, Shanghai 200003, China; ^2^ The Institute of Biomedical Sciences, East China Normal University, Shanghai 200241, China; ^3^ Department of Orthopaedics, The Third Xiangya Hospital of Central South University, Changsha, Hunan 410013, China; ^4^ Department of Molecular Biosciences, Institute of Cellular and Molecular Biology, The University of Texas at Austin, Austin, TX 78712, USA

**Keywords:** EGFR, TKIs, NSCLC, Tumor growth

## Abstract

The epidermal growth factor receptor (EGFR) is a therapeutic target (oncotarget) in NSCLC. Using *in vitro* EGFR kinase activity system, we identified a novel small molecule, WB-308, as an inhibitor of EGFR. WB-308 decreased NSCLC cell proliferation and colony formation, by causing G2/M arrest and apoptosis. Furthermore, WB-308 inhibited the engraft tumor growths in two animal models *in vivo* (lung orthotopic transplantation model and patient-derived engraft mouse model). WB-308 impaired the phosphorylation of EGFR, AKT, and ERK1/2 protein. WB-308 was less cytotoxic than Gefitinib. Our study suggests that WB-308 is a novel EGFR-TKI and may be considered to substitute for Gefitinib in clinical therapy for NSCLC.

## INTRODUCTION

Despite the large number of clinical trials aimed at improving patients’ survival, lung cancer remains the leading cause of cancer-related mortality worldwide in both men and women. Lung cancer is composed of two main types; one type is non-small-cell lung cancers (NSCLCs; 80% of all lung cancers), including adenocarcinomas, squamous cell carcinomas, and large cell carcinomas, and the other type is the small-cell lung cancers, exhibiting neuroendocrine features. According to the latest report, the average 5-year survival rate for NSCLC is only 16% [1]. Over half of lung cancer cases are diagnosed after metastasis, for which the median survival time is approximately 8 months. Approximately 80% of all lung cancer cases are categorized as non-small cell lung cancer (NSCLC), which is typically diagnosed at advanced stages [2, 3]. With regards to lung cancer mortality, 80%–90% of these cases are associated with metastasis and one of the most common cases is bone metastasis [4, 5]. Among lung cancer bone metastasis, the most frequent target organ is the vertebral column, which causes even more severe consequences to patients’ recovery rate and life quality [6, 7].

Growing evidence reveals that epidermal growth factor receptor (EGFR) plays a pivotal role in tumorigenesis, drug resistance, relapse and the metastasis of various cancers [8–11]. As one of the most common cell-surface receptor of the epidermal growth factor family (EGF-family) of extracellular protein ligands, the epidermal growth factor receptor belongs to the ErbB family of receptors [12]. The downstream signaling proteins involved with several signal transduction cascades, such as the MAPK, Akt, ERK1/2 and JNK pathways, are triggered by EGFR autophosphorylation. Additionally, they are associated with tumorigenesis based on their carcinogenic effects on DNA synthesis and properties that promote cell proliferation [13]. Meanwhile, mutations that result in EGFR overexpression or over-activity have also been implicated in a number of cancers, including lung cancer, in particular non-small cell lung cancer [14]. Given that EGFR is one of the most commonly mutated genes in NSCLC [15], EGFR has been considered as an important target for NSCLC therapeutics [16]. Meanwhile, Gefitinib is considered to be the primary selective EGFR tyrosine kinase inhibitor (TKI) for NSCLC therapy, which operates by repression of EGFR oncogenic signaling [17].

Drug resistance to Gefitinib treatment is mostly a result of mutations occurring within the kinase domain of EGFR. Since Paez JG initially reported that EGFR-related mutations alter the effects of drug therapy, additional drug resistant molecular mechanisms have been revealed [17]. Among those, EGFR-TKIs mutations that alter drug sensitivity commonly occur at exons 18–21, in which 49% contain the exon 19 del (Del E746-A750) mutation and 45% of these EGFR mutations contain the point mutation L858R in exon 21 [18]. These two types of EGFR mutations increase the kinase activity of EGFR, thus resulting in increased sensitivity of NSCLC patients to EGFR-TKIs clinical treatment. Furthermore, not all mutations lead to an increase in sensitivity to treatment. For instance, mutations that involve insertions at exon 20 impart resistance to the EGFR-TKIs. Nearly half the drug-resistant NSCLC patients possess the T790M mutation in exon 20 of EGFR, which has been considered the main cause of drug resistance [19]. A secondary mutation at T790M in EGFR has also been associated with drug resistance, as well as Met amplification [20]. Most mutations, except T790M or Met amplification, may be eradicated with the appropriate choice and combinations of second generation tyrosine kinase inhibitors such as Erlotinib and Afatinib. However, there is still no effective TKI available for NSCLC with the T790M and Met amplification mutations. Therefore, it is of great significance to develop new small molecules targeting EGFR that can overcome the chemotherapy resistance to combat lung cancer, especially the NSCLC.

**Figure 1:**
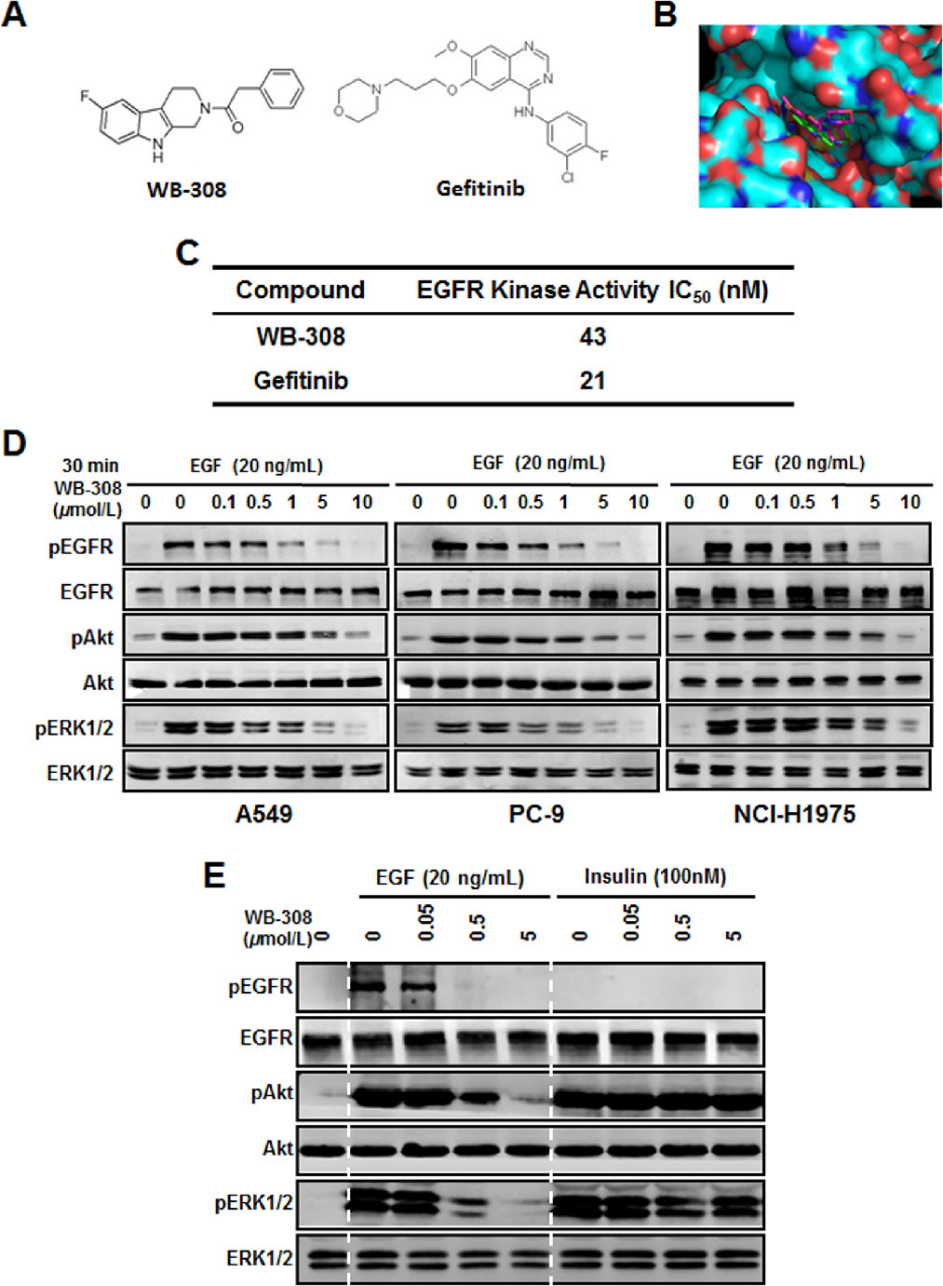
WB-308 suppresses the EGFR signaling pathway in NSCLC cells **(A)** The chemical structure of WB-308 (left) and Gefitinib (right). **(B)** The binding mode of WB-308 and Gefitinib bound with the pocket of the EGFR kinase domain. WB-308 inserts into the binding site of EGFR (right). The binding cavity is shown as achromatic surface. **(C)** *In vitro* EGFR kinase inhibition. Half maximal inhibitory concentration (IC_50_) values of WB-308 and positive control Gefitinib. **(D)** Suppression by WB-308 of EGF–induced phosphorylation of AKT and ERK1/2 in NSCLC cell lines A549, PC-9 and NCI-H1975. Cancer cells were pretreated with indicated concentrations of WB-308 for 4 hours, and then incubated with 20 ng/ml of EGF for another hour. Cell lysates were subjected to Western blotting analysis with indicated antibodies. **(E)** WB-308′s inhibition to the phosphorylation of AKT and ERK1/2 was directly through EGFR but not Insulin receptor. PC-9 cells were pretreated with indicated concentrations of WB-308 for 4 hours, and then incubated with 20 ng/ml of EGF and 100 nM Insulin, respectively, for another hour. Cell lysates were subjected to Western blotting analysis with antibodies as indicated.

In this study, we screened a chemical library of novel compounds synthesized in our lab and identified a novel compound, entitled WB-308 (Figure 1A). WB-308 is a potent inhibitor that is effective against wide type and mutant EGFR kinase activity, and in turn, exhibits impressive anti-cancer activities for NSCLC. Additionally, our results establishes that the unique properties of WB-308 rely on its diminished cytotoxicity when compared to existing EGFR inhibitory compounds, such as Gefitinib.

## RESULTS

### Screening for EGFR signaling inhibitors

For screening of EGFR signaling inhibitors for lung cancer therapy, we downloaded the x-ray crystal structure of EGFR kinase domain from the Protein Data Bank, and Autodock4.2 software package was employed to perform the molecular docking modeling assay. From a compound library of over 50 different compounds for the potential inhibitors of kinase activity of EGFR synthesized in our lab, a novel small molecular weight 308, namely WB-308, was found to be directly bound with EGFR kinase active site in the pocket domain. Figure 1A showed the chemical structure of WB-308 (Figure 1A, left) and Gefitinib (Figure 1A, right) while Figure 1B demonstrated the molecular docking modeling result (Figure 1B). From Figure 1B it could be concluded that the small molecular WB-308 (green), like Gefitinib (amaranth), inserted into EGFR pocket domain, which was located at the kinase active site, thus destroying the kinase activity of EGFR (Figure 1B). Furthermore, both the two small molecules exhibited approximately the same binding energy with EGFR. The above results strongly suggest that EGFR may function as an EGFR inhibitor, thereby serving the same role as Gefitinib.

### Inhibition of EGFR kinase activity of WB-308

According to the aforementioned bio-informatic analysis suggestions, the EGFR kinase assay was carried out in the presence of WB-308 or the well-known EGFR protein kinase inhibitor Gefitinib. As shown in Figure 1C, the kinase activity inhibition IC_50_ for Gefitinib was nearly 21 nM (Figure 1C), which is consistent with a previous report [21]. The inhibition IC_50_ for WB-308 was about 43 nM (Figure 1C), suggesting WB-308 directly inhibited the kinase activity of EGFR at a concentration similar to Gefitinib.

### WB-308 suppresses the EGFR signaling pathway in NSCLC cells

Previous studies revealed that cytokines like EGF could induce the activation of EGFR kinase activity and up-regulate the phosphorylation of downstream signaling pathways, including ERK1/2 and AKT pathways [22]. Herein we employed 5 different NSCLC cells, including A549, PC-9, NCI-H1975, NCI-H1650, and HCC-827 to test the effect of WB-308 on EGF-induced EGFR phosphorylation by Western blot analysis. A549 exemplifies the wild type EGFR kinase prototype, while PC-9 possesses the 19 del (Del E746-A750) mutation of EGFR, and NCI-H1975, NCI-H1650, and HCC-827 all contain EGFR with T790M and L858R mutations.

Our data showed that EGF (20 ng/mL for 30 min) strongly stimulated the phosphorylation of EGFR protein in 5 NSCLC cells while WB-308 reduced EGF-induced EGFR phosphorylation in a dose-dependent manner in these NSCLC cell lines (Figure 1D for A549, PC-9, NCI-H1975 and S1A for NCI-H1650 and HCC-827). And WB-308 at 10 μM fully blocked EGFR phosphorylation. Meanwhile, after the treatment of EGF at 20 ng/mL for 30 min, the phosphorylation levels of AKT and ERK1/2 decreased accordingly in the same manner as p-EGFR, while the total amount of these signaling proteins were not affected (Figure 1D and Figure S1A). These results confirm the fact that WB-308 inhibited EGFR and its downstream signaling pathways.

To further confirm that WB-308′s inhibition to the phosphorylation of AKT and ERK1/2 was directly through EGFR, but not through other cytokine receptor pathways, we conducted the same experiments using EGFR and Insulin, both in PC-9 cells. Insulin did not serve as the ligand for EGFR and would not induce the phosphorylation of EGFR, but it could cause the phosphorylation of AKT and ERK1/2 [23]. As showed in Figure 1E, after the stimulation of EGF (20 ng/mL), the phosphorylation of EGFR and its’ downstream signaling proteins remarkably increased and WB-308 significantly inhibited this increase in a dose-dependent manner. However, in the presence of Insulin (100 nM), although the phosphorylation level of EGFR stood steadily, the phosphorylation levels of AKT and ERK1/2 were up-regulated obviously. Furthermore, pre-treatment of WB-308 failed to inhibit the phosphorylation of AKT and ERK1/2 (Figure 1E). Taken together, all these results indicate that WB-308 impaired the phosphorylation of EGFR as well as the phosphorylation of many EFGR signaling pathway proteins in NSCLC cells, therefore it can be identified as an novel small molecular EGFR inhibitor.

### WB-308 exerts the same molecular mechanism with Gefitinib in EGFR mutated NSCLC cells

**Figure 2:**
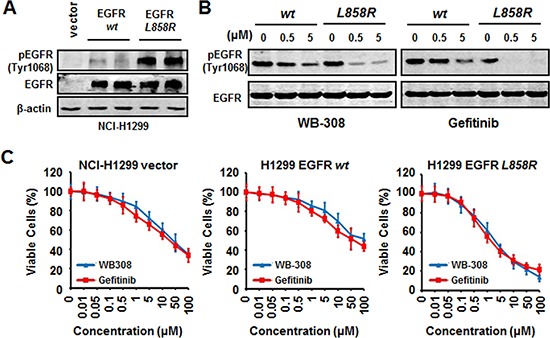
WB-308 exerts the same molecular mechanism with Gefitinib in EGFR mutated NSCLC cells **(A)** Establishment of the EGFR-WT, EGFR-L858R and the vehicle over-expressing stable lines. The 3 stable cell lysates were subjected to Western blotting analysis with antibodies against p-EGFR (Tyr1068) and EGFR. Anti-β-actin antibody (1:10 000 dilution) was used as a loading control. **(B)** WB-308 increases NSCLC cell's sensitivity EGFR mutation. H1299 (EGFR-WT) and H1299 (EGFR-L858R) stable cell lines were exposed to indicated concentrations of WB-308 for 4 hours and cell lysates were subjected to Western blotting analysis with antibodies against p-EGFR (Tyr1068) and EGFR. **(C)** WB-308 suppresses NSCLC cell's proliferation in EGFR mutation. The cell viability assay stained by SRB was performed as described in Materials and Methods. Columns, mean; bars, SE (*n* = 3; *t*-test, *P* < 0.05).

It has been previously reported that EGFR-TKIs drug sensitivity-related mutations commonly occur at exons 18–21, 49% of which are exon 19 del (Del E746–A750) mutations, and 45% of which contain the L858R mutation at exon 21. When these two types of EGFR mutations are present, NSCLC cell's sensitivity to clinical chemotherapy drugs, including Gefitinib, increases [24]. To confirm that WB-308 operates via the same mechanism as Gefitinib in response to EGFR mutations, we employed a de novo system to test WB-308′s working model in wild type NSCLC cell line H1299 in order to exclude artificial results and background. Considering the mutant efficiency and experiment simplicity, we chose the L858R mutation to confirm our result because to construct a point mutant is much easier that to construct a deletion mutant. We established stable cell lines that could stably express WT EGFR and L858R-mutated EGFR by transfecting with plasmid constructs in NCI-H1299 cells, which is an EGFR low-expressed cell line. The empty vector plasmid was employed as negative control in this experiment. Western blotting analysis demonstrated that these 3 stable cell lines, namely H1299 (vector), H1299 (EGFR–WT), and H1299 (EGFR–L858R), were successfully established (Figure 2A). Next we treated these cells with WB-308 and Gefitinib, respectively. Within our expectations, WB-308 showed the same effects as Gefitinib, further emphasizing the same effective model, and inhibited the phosphorylation of EGFR–L858R more than that of EGFR-WT, in a dose dependent manner. At a concentration of 0.5 μM, WB-308 decreased the level of p-EGFR of EGFR-L858R stable cells even more severely than that in EGFR-WT stable cells at the concentration of 5 μM, which indicated that the NSCLC cell's sensitivity to WB-308 increased after EGFR mutation occurred (Figure 2B).

Provided the model that WB-308 inhibits EGFR signaling pathway to different degrees in the EGFR-WT cell and EGFR-L858R cell, we examined whether WB-308 suppressed NSCLC cell proliferation to different degrees using the SRB assay, too. As shown in Figure 2C, like Gefitinib, WB-308 had inhibitive effects on H1299 (vector), H1299 (EGFR-WT), and H1299 (EGFR–L858R) cells, however, the inhibition effect to H1299 (EGFR–L858R) cell was the most dramatic. For example, at a concentration of 5 μM, the percentages of cell viability of H1299 (vector) and H1299 (EGFR-WT) were about 80%, while that of H1299 (EGFR–L858R) dramatically decreased to 40% (Figure 2C), which further confirmed that like Gefitinib, NSCLC cell's sensitivity to WB-308 also increased after the EGFR L858R mutation was present. In summary, WB-308 operates via a similar molecular mechanism as Gefitinib, and could be considered a substitute for Gefitinib.

### WB-308 exhibits evidences to be a better EGFR inhibitor than Gefitinib

**Figure 3:**
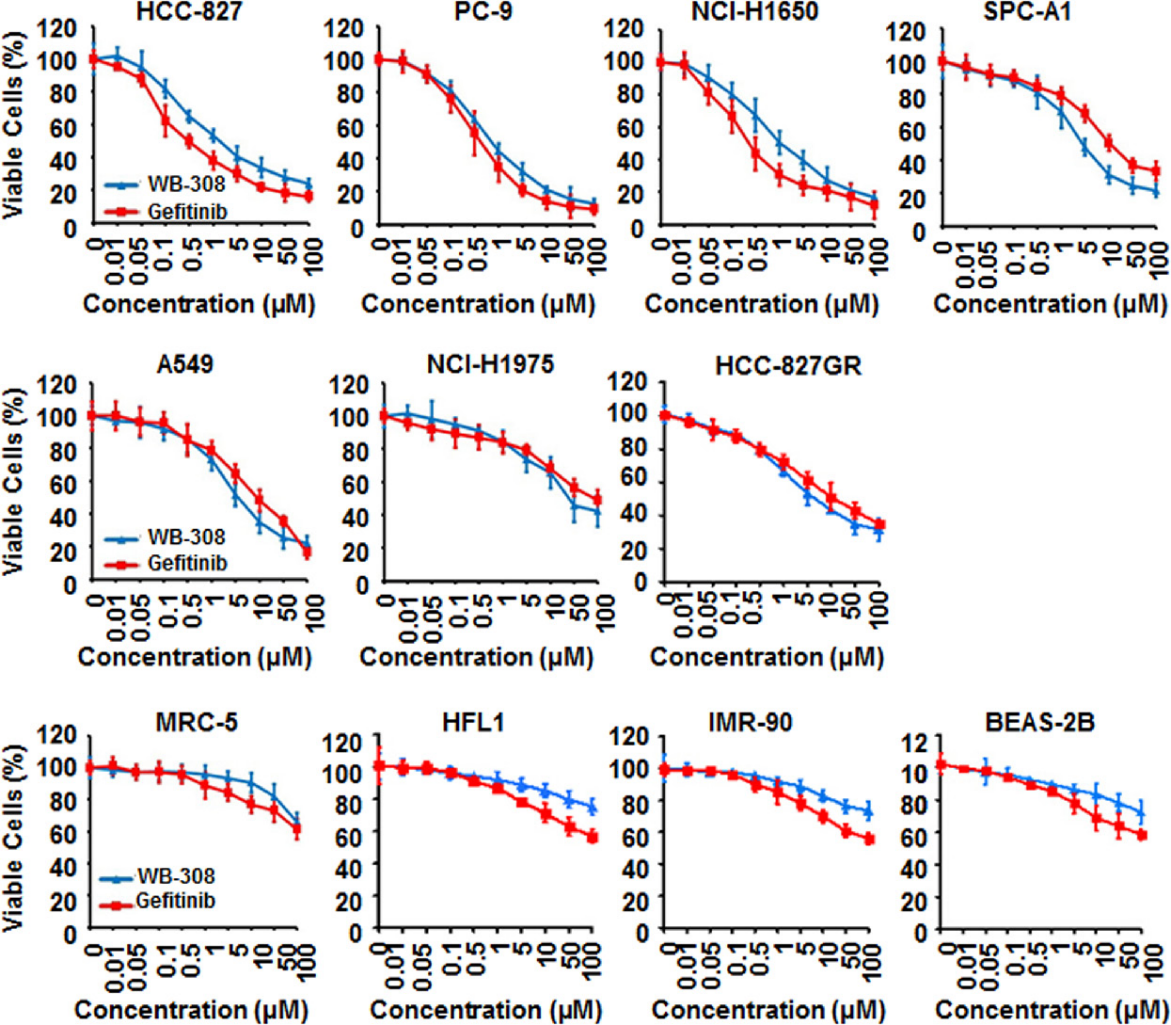
WB-308 exhibits evidences to be a better EGFR inhibitor than Gefitinib 11 NSCLC cell lines (HCC-827, PC-9, NCI-H1650, SPC-A1, A549, NCI-H1975, HCC-827GR, MRC-5, HFL1, IMR-90 and BEAS-2B) were subjected to the cell viability assay stained by SRB as described in Materials and Methods. Columns, mean; bars, SE (*n* = 3; *t*-test, *P* < 0.05).

The aforementioned results suggest that WB-308 inhibits the proliferation of NSCLC cells. Furthermore, we questioned if WB-308 had ideal selective effects between NSCLC cells and healthy human lung tissue cells, such as the human lung fibroblast cell MRC-5, and the normal human lung epithelial cells including HFL1, IMR-90 and BEAS-2B, compared to Gefitinib. Eleven NSCLC cell lines, including HCC-827, PC-9, NCI-H1650, SPC-A1, A549, NCI-H1975 and HCC-827GR, together with the human normal lung tissue cells MRC-5, HFL1, IMR-90 and BEAS-2B, were utilized to conduct the cell viability assay. A549 and SPC-A1 represent the wild type EGFR while in PC-9 the 19 del (Del E746–A750) mutation of EGFR occurs, and NCI-H1975, NCI-H1650, and HCC-827 all three possess the T790M mutation of EGFR. HCC-827GR cells contain Met amplification after the T790M mutation occurred. SRB assay results clearly showed that the half maximal inhibitory concentration for WB-308 inhibition of NSCLC cellular proliferation was approximately 5 μM, while that of 4 human normal lung tissue cells were much more than 100 μM (Figure 3). In short, WB-308 was more toxic to NSCLC cells than to normal lung tissue cells. Meanwhile, WB-308 at all concentrations caused less cytotoxicity to 4 human normal lung tissue cells than did Gefitinib (Figure 3 for MRC-5, HFL1, IMR-90 and BEAS-2B), which suggested that WB-308 had higher levels of bio-safety than Gefitinib. Although in these 6 EGFR-WT or EGFR-mutated cells, WB-308 showed comparable effects to Gefitinib, regardless of cell type. In each of EGFR-WT cells (SPC-A1 and A549), EGFR T790M mutant cells (NCI-H1975), or in Met-amplification cells (HCC-827GR), WB-308 caused severe inhibition effects when compared with Gefitinib (Figure 3), indicating that WB-308 should have less toxicity. Therefore, it can be considered that WB-308 has unique properties compared to the existing EGFR inhibitor Gefitinib and may serve as a better EGFR inhibitor than Gefitinib.

### WB-308 inhibits colony formation of NSCLC cells

**Figure 4:**
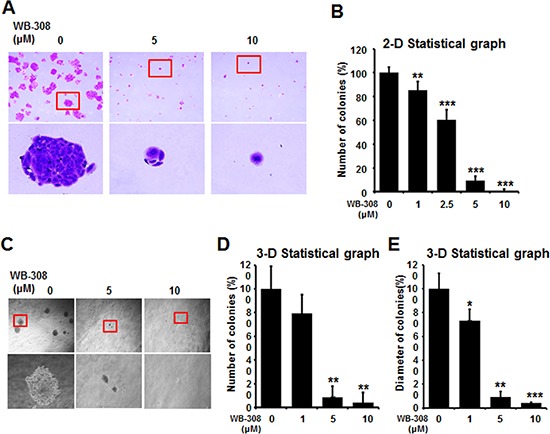
WB-308 inhibits colony formation of NSCLC cells **(A)** NSCLC cell line SPC-A1 was seeded in 6-well plates for 7 days after the treatment of WB-308 in according concentrations and fixed with 4% paraformaldehyde, and stained with 0.2% crystal violet. **(B)** Statistic results of 2-D colony formation. Columns, mean; bars, SE (*n* = 3; *t*-test, *P* < 0.05). **(C)** NSCLC cell line SPC-A1 was subjected to the 3-D colony formation assay described in Materials and Methods and the result were photographed. **(D, E)** Statistic results of 3-D colony formation. Columns, mean; bars, SE (*n* = 3; *t* test, *P* < 0.05).

Two kinds of colony formation assays were performed to test the inhibition effect of WB-308 to NSCLC cell proliferation. One was the 2-D colony formation assay and the other was the 3-D colony formation assay. As Figure 4 shows, WB-308 inhibited colony formation of NSCLC cells in a concentration-dependent manner, and showed a very significant difference compared to the control group when at 10 μM. In Figure 4A, after being seeded in 6 cm dishes and colony formatted for 2 weeks, SPC-A1 cells displayed a decreased number of colonies with the increase of WB-308 concentration (Figure 4A, the upper panel). The bottom panel of Figure 4A illustrates the enlarged view of the respective colony. Figure 4B, 4D and 4E show the statistic results of each colony formation assay, according to the diagram of colony numbers and colony diameters. The 3-D colony formation morphology results are demonstrated in Figure 4C. From Figure 4 we can summarize that WB-308 inhibits colony formation of NSCLC cells.

### WB-308 induces NSCLC cell apoptosis and arrests NSCLC cells at G2/M phase

**Figure 5:**
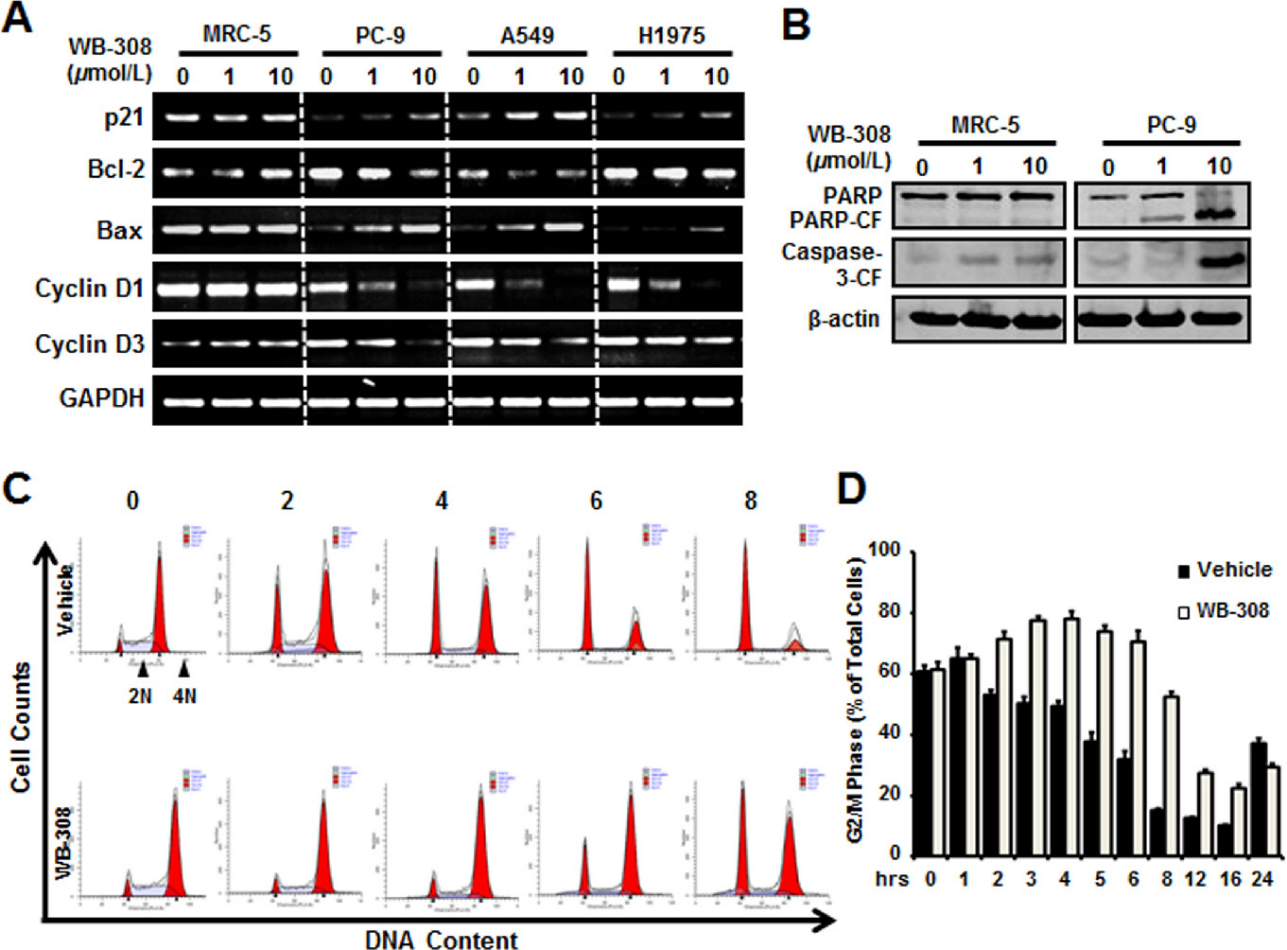
WB-308 induces NSCLC cell apoptosis and arrests NSCLC cell at G2/M phase **(A)** Five representative genes with expression levels after the treatments of indicated concentrations of WB-308. Effects of WB-308 on transcription of five cell apoptosis and cell cycle checkpoint-associated genes were analyzed through the real-time PCR. **(B)** MRC-5 and PC-9 cell lines were exposed to indicat concentrations of WB-308 for 4 hours and cell lysates were subjected to Western blotting analysis with indicated antibodies. Anti-β-actin antibody was used as a loading control. **(C)** Cell cycle analysis of PC-9 cells. PC-9 cells were treated with Nocodazole (100 ng/ml) for 16 hrs aiming to synchronize cell cycle at the same phase and then release, then cells were exposed to different concentration of WB-308 for according time. After PI staining, cells were analyzed by flow cytometry. **(D)** Statistic results of cell cycle arrested in G2/M phase. Columns, mean; bars, SE (*n* = 3; *t*-test, *P* < 0.05).

To further identify whether WB-308 could induce apoptosis and cell cycle arrest, we performed real-time PCR analysis by probing the expression levels of the following genes: p21, Bcl-2, Bax, Cyclin D1, and Cyclin D3, all of which are typical markers related with cell apoptosis and cell cycle checkpoints. As shown in Figure 5A, WB-308 had little effect on these typical proteins’ mRNA levels in MRC-5 cells, but had tremendous influences on PC-9, A549, and H1975 cells. The mRNA levels of p21, Bcl-2, Cyclin D1 and Cyclin D3 were down-regulated while that of Bax were upregulated (Figure 5A). Meanwhile, we immunoblotted the protein levels of apoptosis marker genes, such as PARP and Caspase-3. Figure 5B demonstrates that our hypothesis was proven correct. In PC-9 cells, the cleaved PARP and cleaved Caspase-3 protein levels correlated directly to increasing concentrations of WB-308. However, in MRC-5 cells, the situation was different (Figure 5B). Next we examined the effects of WB-308 on the cell cycle through analysis by flow cytometry. Nocodazole treatment was performed for cell cycle synchronization and subsequent release, and then we tracked the cell cycle distribution at different time points. Results showed that WB-308 remarkably changed the cell cycle, when compared with the vehicle control group (Figure 5C). Statistically, we found that WB-308 increased cell numbers at G2/M phase from about 60% to nearly 80% as its release time increased from 0 hr to 5 hrs in PC-9 cell while the vehicle group didn’t increase but instead decreased (Figure 5D). In short, our data elucidated that WB-308 induced cell apoptosis and arrested the cell cycle at G2/M phase.

### WB-308 inhibits PC-9 orthotopic tumor growth *in vivo*

**Figure 6:**
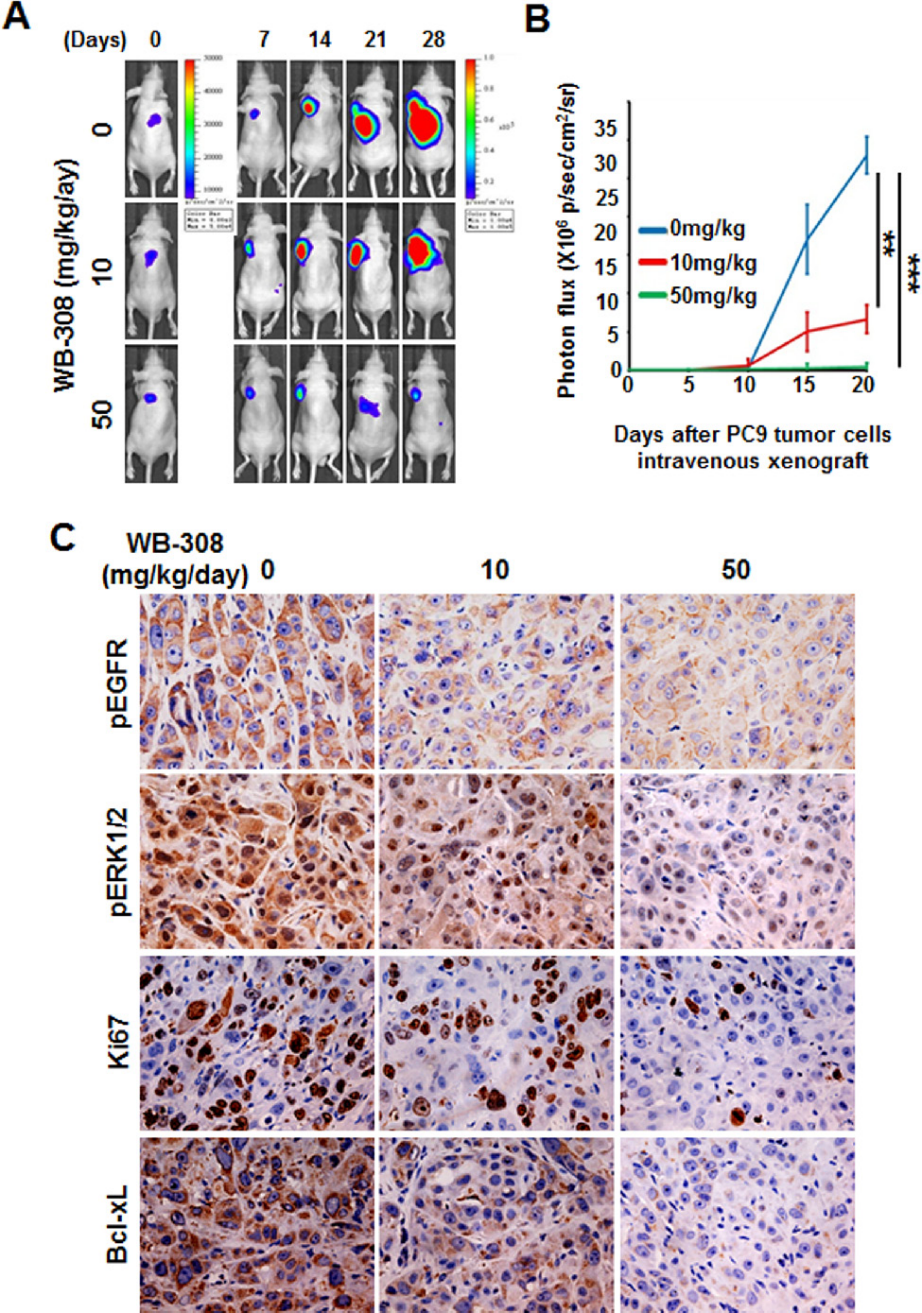
WB-308 inhibits PC-9 orthotopic tumor growth *in vivo* **(A)** Tumor growth in the orthotopic lungs over a 28-day period was detected by bioluminescence analysis every 7 days. **(B)** Quantitative analysis of growing cells in lung bioluminescence analysis every 5 days. The means and 95% confidence intervals (error bars) are presented; ***P < 0.001, **P = 0.004. *P* values were calculated using a two sided Student's *t*-test. p/sec/cm^2^/sr = photons/second/cm^2^/steradian. **(C)** To detect the inhibitory effect of WB-308 on tumor cell proliferation and EGFR signaling pathway in PC-9-luc orthotopic tumor growth model, primary tumors and lungs were sectioned and probed with anti-pEGFR (1:100 dilution), anti-pERK1/2 (1:200 dilution), anti-Ki-67 (1:200 dilution) and anti-Bcl-xL (1:100 dilution) antibodies. Representative images are shown. Brown color indicates positive cells. Scale bar = 30 μm.

The *in vivo* effects of WB-308 on NSCLC were rigorously tested using animal models. First, we evaluated the inhibitory effect of WB-308 on PC-9 orthotopic tumor growth model *in vivo*. Initially, we constructed a luciferase-expressing PC-9 cell line (PC-9-luc). Upon orthotopic injection into mice lungs from day 0–28, PC-9-luc cells exhibit bioluminescence which is traced by photon flux indexes, in turn representing the tumor sizes with use of the IVIS 2000 Luminal Imager system. Mice were divided into 3 groups (*n* = 10 per group) and treated with WB-308 at 10 mg/kg/day or 50 mg/kg/day or vehicle control. In the control mice, tumor cells were detected in the lung regions with high levels of bioluminescence, which increased remarkably with day number increase (Figure 6A, the upper panel). Treatment with WB-308 statistically reduced the photo flux indexes (Figure 6A, the middle and the bottom panel, Figure 6B). As Figure 6B showed, at day 28 the nude mice were sacrificed. The average normalized photon flux of the 10 mg/kg/day WB-308 treated group and 50 mg/kg/day WB-308 treated group was 8 × 10^6^ ± 0.76 p/sec/cm^2^/sr and 0.18 × 10^6^ ± 0.03 p/sec/cm^2^/sr, respectively, while that of vehicle control group was 28 × 10^6^ ± 0.32 p/sec/cm^2^/sr (Figure 6B). Moreover, no statistically significant difference in mouse body weight was detected among these three groups, suggesting low compound toxicity. We also dissected mice following sacrifice, and the anatomy results showed no obvious pathological effect on the main organs (data not shown). Together, our data indicates that administration of WB-308 therapeutically blocked NSCLC tumor growth.

Western blot results shows that compared with control mice tumors, the phosphorylation of EGFR and ERK1/2 decreased in WB-308-injected mice tumors in a dose-dependent manner (Figure 6C), suggesting that WB-308 could suppress the EGFR pathway *in vivo*. Similar results were obtained from the analysis of Ki-67 and Bcl-xL expression, which were well-known classical tumor cell proliferation markers (Figure 6C). Taken together, these *in vivo* immunohistochemistry results were in agreement with our *in vitro* results and indicated that WB-308 suppressed NSCLC tumor growth by suppressing the EGFR signaling pathway.

### WB-308 inhibits the growth of patient-derived NSCLC tumor xenografts

**Figure 7:**
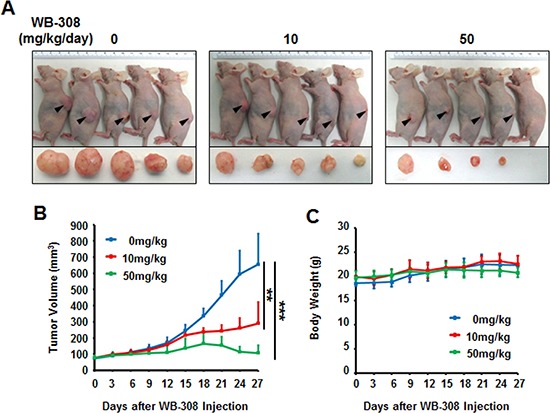
WB-308 inhibits the growth of patient-derived NSCLC tumor xenografts **(A)** The patient-derived spinal metastasized tumor cells were injected subcutaneously into the nude mice. The tumors model was established according to the steps described in Materials and Methods. After mice sacrificed, tumors were removed and images taken with a Nikon camera. **(B)** Statistic results of quantitative analysis of growing tumor volume in mice back subcutaneous every 3 days. The means and 95% confidence intervals (error bars) were presented (***P < 0.001, **P = 0.004). **(C)** Effect of WB-308 on mouse body weight. WB-308 did not affect the body weight of mice when recorded every 3 days. Columns, mean (*n* = 10); bars, SE. **P < 0.01 versus control.

Next, we clinically examined the effect of WB-308 on NSCLC tumors. It has been widely accepted that patient-derived tumor xenograft models can be utilized as an ideal drug-screening tool for many kinds of cancer therapy [25–27], including therapy of lung cancer [28]. Lung cancer usually causes bone metastases, especially the vertebral metastasis. Thus, we employed a patient-derived NSCLC cell line, which was primarily separated from an advanced NSCLC patient's spinal metastasis in Changzheng Hospital (Shanghai, China), to test if WB-308 could be clinically beneficial. First, the SRB assay was performed to identify the effects of WB-308 on this patient-derived NSCLC cell line. As shown in supplymentary Figure 1B, WB-308 inhibited the cell proliferation of this cancer cell line in a dose-dependent manner, and Gefitinib was used here as a positive control (Figure S1B). Then, we injected this cancer cell line into nude mice to establish the patient-derived NSCLC tumor xenograft model. Mice were divided into 3 groups (*n* = 10 per group) and treated with WB-308 at 10 mg/kg/day or 50 mg/kg/day or vehicle control. At the day 27, mice were sacrificed and the tumor xenograft of each mouse was dissected (Figure 7A). The average tumor volume of control group was 654 ± 190 mm^3^, whereas tumor size in WB-308-treated group was 289 ± 132 mm^3^ for 10 mg/kg/day group and 107 ± 46 mm^3^ for 50 mg/kg/day group, respectively. And statistical results showed a significant difference between the drug-treated groups and the control group (Figure 7B), especially for the 50 mg/kg/day group, the tumor burden of each mouse almost ceased to grow following the administration of WB-308 (Figure 7B). At the same time, treatment of WB-308 at the given concentration still had little effect on the body weights of the WB-308-treated mice, when compared to the control group (Figure 7C), which further confirmed that WB-308 had low toxicity to mice at the curative dose.

## DISCUSSION

The EGFR signaling pathway has been shown to promote tumor progression in many types of human cancers [29]. EGFR plays a critical role in regulating cancer cell growth and survival, representing an attractive therapeutic target in NSCLC and compounds serve as EGFR-TKIs are attracting even more and more attention [30]. The first-generation EGFR-TKIs, such as Gefitinib, have proven to be highly effective for advanced NSCLC. Although these small molecular targeted agents provide a significant response and survival benefit, all responders eventually acquire chemotherapy resistance [31]. What's more, when considering the clinical benifits of Gefitinib, the assessment with evidence of the efficacy and toxicity of the treatment of NSCLC is one of the most capital factors restraining its application in the course of treatment [32]. Till now, many reports focus on both efficacy and toxicity of the current state of Gefitinib therapy in NSCLC patients, and some typical adverse effects have been revealed [33, 34]. Unanswered questions about the optimal use of Gefitinib still remain open.

To date, most of the known small molecular EGFR-TKIs are anilinoquinazoline chemotypes, including Gefitinib and Erlotinib. The primary objection of our research is to expand the candidate scope of leading compounds to substitute existing EGFR-TKIs, especially Gefitinib. In this study, we identified a novel small molecule inhibitor, WB-308, which targets the kinase activity of EGFR and EGFR-mediated tumor growth-associated gene expression. Moreover, WB-308 suppressed NSCLC cancer cell proliferation, cell viability, and colony formation ability, and induced cell apoptosis and cell cycle arrest in a dose-dependent manner. In two different mouse tumor growth models, we also found that WB-308 therapeutically blocked NSCLC tumor growth by suppressing the EGFR signaling pathway. In addition, the maximum effective concentration of WB-308 (50 mg/kg/day) did not induce apparent toxicity to mice. Functionally, WB-308 shows the same anti-cancer activities as Gefitinib. However, upon comparing the balance between efficacy and toxicity, the most significant benefit of WB-308 over Gefitinib is that WB-308 exerts less cytotoxicity, as observed upon treatment of the four primary normal human lung tissue cell lines, MRC-5, HFL1, IMR-90 and BEAS-2B. WB-308 also possesses a novel chemical backbone structure, which is substantially different from known typical EGFR-TKIs, which provides a new structural scaffold for future modification and development of potential anti-cancer agents against NSCLC.

Various mechanisms of drug resistance have been identified in NSCLC for EGFR-TKIs treatment. Small in-frame deletions at exon 19 and point mutations within exon 21 (L858R) are the most common EGFR-activating mutations, which both lead to sustained kinase activity. The EGFR-T790M secondary mutation is responsible for half of the cases of acquired resistance to EGFR-TKIs and Met amplification, which permits cell survival by persistent activation of Akt signaling, has been described for 5 to 15% of cases [20]. Upon WB-308 treatment of different cell lines that harbor different types of EGFR mutants, all lines reacted in the same manner to Gefitinib. This result indicates that WB-308 has the same efficacy and can be regarded as an ideal substitute for Gefitinib. Furthermore, WB-308′s low toxicity suggests that WB-308 might overcome the negative implications of Gefitinib, allowing it to be a beneficial alternative candidate for Gefitinib in clinic.

As for NSCLC, about 22%–64% of advanced lung cancer patients will eventually develop bone metastasis [35]. In this study, the patient-derived xenograft model suggested that WB-308 may exert its function in NSCLC patients. Because the cancer cells were derived from patients’ spine metastatic tumors, they mimic the process of cancer cell colony formation and migrate to target bones *in vivo*. WB-308 functions sufficiently in the NSCLC patient-derived xenograft model, thus may provide evidence for its future clinical applications to cure NSCLC bone metastasis patients.

However, this study still has a few limitations, and further comprehensive development of the WB-308 model is needed. Initially, we have only examined the influence of WB-308 on the Ras/Raf/MEK/ERK-MAPK signaling pathway and the PI3K-AKT-mTOR signaling pathway, and have yet not to acknowledge additional pathways, such as the Src-JAK-STATs signaling pathway and the PLCγ-PKC signaling pathway, all of which are downstream of EGFR pathways and associated with tumor growth. In order to fully understand the molecular mechanism of WB-308 on the EGFR pathway, it is necessary to ascertain whether WB-308 further influences additional EGFR downstream signaling pathways. Secondly, in the two different tumor growth mouse models, we treated the mice only by intraperitoneal injection, not oral or intravenous application of WB-308. The drug metabolism, pharmacokinetics, and pharmacodynamics of this compound need to be further investigated to determine whether the amount of absorbed WB-308 is enough to function in NSCLC patients. Meanwhile, the indices of WB-308′s solubility, stabilization and biological availability are all needed to be carefully confirmed. Thirdly, when performing the animal model experiments, we were not blind to the DMSO-treated control mice and WB-308-treated mice, which could lead to biased interpretation of the results. A randomized double-blind controlled trial of WB-308 on animal model experiments is going to be necessary. Finally, considering the role of the EGFR signaling pathway in numerous human cancer symptoms—for example, cancer metastasis, tumor invasion and angiogenesis (not including tumor growth), [29] as well as other types of tumors, including lung cancer [36], breast cancer [37], prostate cancer [38], colon cancer [39], gastric cancer [40], glioma [41], melanoma [42], *etc*.—it is reasonable to speculate whether WB-308 may have effects on EGFR signaling pathway-related human cancer types. Careful evaluation will be further required in these other EGFR-related cancer types. Regardless, to firmly establish the clinical significance of WB-308, more detailed tests are further needed.

In conclusion, our current preclinical study of WB-308 demonstrated that WB-308, a novel small molecule inhibitor, functions similarly to Gefitinib, and impairs the kinase activity of EGFR, thus inhibiting human NSCLC cell growth. Furthermore, mouse models have indicated that systemic administration of WB-308 prevents the lung cancer cell xenograft model, especially the patient-derived xenograft mouse model. Additionally, WB-308 is unique for its diminished cytotoxicity levels when compared to Gefitinib. We therefore conclude that WB-308 may be considered an ideal substitute for Gefitinib and have potential applications for clinical treatment in NSCLC therapy.

## MATERIALS AND METHODS

### Cells, reagents and animals

Human NSCLC cells HCC-827, NCI-H1650, SPC-A1, A549, NCI-H1975, HCC-827GR, human normal lung fibroblast cell MRC-5, and normal human lung epithelial cells HFL1, IMR-90 and BEAS-2B, and the Luciferase-labeled human NSCLC PC-9 cell line were all purchased from American Type Culture Collection. The patient-derived NSCLC cell line was primarily separated from an advanced NSCLC patient's spinal metastasis in the department of orthopedic oncology of Changzheng Hospital (Shanghai, China), with the patient's informed consent. Recombinant human EGF and Insulin were purchased from PeproTech, Inc. (Rocky Hill, NJ). Kinase-Glo Luminescent Kinase Assay kit was purchased from Promega (Promega, Mannheim, Germany). Gefitinib was purchased from Shanghai Alis Chemicals Co. Ltd. The small molecular WB-308 and other moleculars in the chemical library were synthesized in our laboratory. Antibodies for phospho-EGFR (Tyr1068) (#3777), EGFR (#4267), Phospho-AKT (Ser473) (#4060), AKT (#9272), Phospho-ERK1/2 (Thr202/Tyr204) (#4370), ERK1/2 (#9102) were all ordered from Cell Signaling Technology (Danvers, MA). Antibodies for β-actin (#A5441) was from Sigma-Aldrich Co (St. Louis, MO). Ltd. Antibodies against PARP, cleaved PARP, Caspase 3 and cleaved Caspase 3 were all purchased from Santa Cruz. Nude mice were bred and maintained at the animal center in East China Normal University of Biomedical Sciences and School of Life Sciences of East China Normal University approved all experimental protocols.

### Molecular docking modeling assay

The X-ray crystal structure of EGFR was obtained from the Protein data bank (PDB) website (http://www.rcsb.org/). The structures of the ligands were built and energy minimized using the Chemoffice software package (Cambridge). We used Autodock4.2 version developed by the Scripps Research Institute and Olson lab for free for docking experiments. All of the water molecules were removed before the experiments so that our experiments were performed under non-aqueous conditions. The primary ligand bound to the binding pocket was the chosen conformation for the active site. The picture was prepared using Pymol 1.2R2 version.

### *In vitro* EGFR kinase assay

The half maximal concentration (IC_50_) values of WB-308 and positive control Gefitinib against EGFR kinase activity were carried out using the Promega Kinase-Glo kit (Promega, Mannheim, Germany) according to the manufacturer's protocol in the presence of 600 nM ATP. Data were presented as means and 95% confidence intervals (CIs) from three independent experiments.

### Western blotting analysis

Cells were lysed with radioimmuno-precipitation (RIPA) buffer [(50 mmol/L Tris–HCl (pH 7.4), 150 mmol/L NaCl, 5 mmol/L EDTA, 1% Triton X-100, 1% sodium deoxycholic acid, 0.1% SDS, 2 mmol/L phenylmethylsulfonyl fluoride (PMSF), 30 mmol/L Na2HPO4, 50 mmol/L NaF, 1 mmol/L Na3VO4, and protease or phosphatase inhibitor cocktail (Sigma-Aldrich, Inc. Shanghai, China)]. For xenografts, tumor issues well minced, and lysed with RIPA buffer. Samples were resolved on 8–12% SDS-PAGE and transferred onto polyvinylidene difluoride nitrocellulose membranes (Millipore, Billerica, MA). Membranes were incubated in 5% (w/v) bovine serum albumin (TBST/BSA) and stored overnight at 4°C on a shaker with specific primary antibodies (1/1,000 in TBST/BSA). Membranes were washed with TBST and incubated for 1 h with secondary antibody (1/10,000 in TBST/BSA) at room temperature. After several washes, the signals were visualized via the Odyssey western blot detection system.

### Semi-quantitative real-time polymerase chain reaction analyses

The RNA isolation and semi-quantitative real-time polymerase reaction were performed as described previously [43]. The following primers and probes were used:

Bcl-2-For: 5′-GTGGAGGAGCTCTTCAGGGA-3′
Bcl-2-Rev: 5′-AGGCACCCAGGGTGATGCAA-3′
Bax-For: 5′-GACGAACTG GACAGTAACATG-3′
Bax-Rev: 5′-AGGAAGTC CAATGTCCAGCC-3′
Cyclin D1-For: 5′-GCTGCGAAGTGGAAACCATC-3′
Cyclin D1-Rev: 5′-CCTCCTTCTGCACACATTT GAA-3′
Cyclin D3-For: 5′-TACCCGCCATCCATGATCG-3′
Cyclin D3-Rev: 5′-AGGCAGTCCACTTCAGTGC-3′
GAPDH-For: 5′-ACCCAGAAGACTGTGGATGG-3′
GAPDH-Rev: 5′-TTCAGCTCAGGGATGACCTT-3′

### SRB cell viability assay

SRB cell viability assays were performed by stained with Sulforhodamine B. Briefly, 5000 cells per well were seeded in 96-well plates. After 24 h, cells were exposed to Gefitinib or different concentrations of WB-308 for 72 h. Cells were fixed with 10% trichloroacetic acid for 1 h at 4°C, washed five times with flowing water, and air-dried. Cells were stained with 50 μL 0.4% (w/v) SRB for 20 min at room temperature, washed five times with 1% acetic acid, and air-dried. 100 μL 10 mM Tris was added per well, and absorbance was measured at 515 nm.

### 2-D colony formation assay

Cells were trypsinized and seeded 2000 per well in 6-well dishes. Cells were allowed to attach overnight and then exposed to Gefitinib or different concentration of WB-308 for 7 days. After being fixed with 4% paraformaldehyde for 20 minutes at room temperature, cells were stained with 0.2% crystal violet. The morphology of cell colonies was recorded with photo imaging and the number of cell colonies were calculated and analyzed as the ratio of the number of treated samples to untreated sample. Triplicate wells were set up for each concentration.

### 3-D colony formation assay

Cells were trypsinized and seeded at a concentration of 2000 per well in 6-well dishes that were pretreated with the mixture of media and agarose with the volume ratio of 1:1. Additionally, the mixture also contained Gefitinib or different concentration of WB-308. The cells were cultivated in this media for 3 weeks. After the colonies grew to a suitable volume, the morphology of cell colonies was taken with photo imaging and the numbers of cell colonies were calculated and analyzed as the ratio of the number of treated samples to untreated sample. Triplicate wells were set up for each concentration.

### Cell cycle distribution analysis

Cells were seeded in 6 cm dish initially. And 24 h later, cells were treated with Nocodazole (100 ng/ml) for 16 hrs aiming to synchronize cell cycle at the same phase and then release, then cells were exposed to Gefitinib or different concentration of WB-308 for accordingly time. After being washed with PBS and digested with trypsin, adherent and floating cells were collected, washed once with PBS, fixed in cold 70% ethanol overnight in 4°C. After ethanol fixation, cells were washed once in PBS and resuspended in PBS with 200 μg/ml RNAase and 50 μg/ml propidium iodide (PI) in the dark for 30 minutes. Then cells were then analyzed by flow cytometry (FACS Calibur; BD Biosciences).

### Establishment of stably transfected cell

The EGFR-WT, EGFR-L858R and the vehicle over-expressing plasmids were purchased from Clontech. Generally, the full length EGFR-WT and EGFR-L858R cDNA were cloned into the vehicle vector pcDNCA3.1 to generated EGFR-WT and EGFR-L858R over-expression plasmids. NCI-H1299 cells was transiently transfected with the above 3 plasmids using Lipofectamine 2000 according to the manufacturer's instructions (Invitrogen, MD) and retained in medium containing 10% FBS and 1 μg/ml puromycin to select the stable transfected cells.

### PC-9-luciferase cell orthotropic transplantation xenograft model

PC-9-Luciferase cells (5 × 10^5^) in 0.02 mL liquids that were mixed with PBS and Matrigel were orthotopically transplantated into the lung of nude mice after anaesthetized by pentobarbital sodium. Seven days later, based on photon flux indexes detected by Xenogen IVIS 2000 Luminal Imagier, all mice bearing tumor were devided into three groups (*n* = 10 per group) randomly and from that day on, the luminal photos were taken every 7 days and photon flux indexes which could represent the orthotopic tumor sizes were recorded every 5 days. WB-308 (10 mg/kg/day per mouse and 50 mg/kg/day per mouse) was injected intraperitoneally every day. The control group was treated with dimethyl sulfoxide. 28 days later, the mice were sacrificed, and tumors in lungs were removed and images were recorded. The growth rate curve of the tumor xenograft was evaluated by determining the photon flux indexes. The mouse body weight was measured every 3 days. Lung tumor xenografts were fixed and prepared for immunohistochemistry.

### Patient-derived xenograft model

This assay was performed as described previously with few modifications [44]. Briefly, the patient-derived cells were injected subcutaneously on the right back sides of the mice (7 × 10^6^ cells per mouse). After the tumors reached about 100 mm^3^, we removed them from the mice and dissected them into 30 little pieces, equally. Then these 30 little tumor pieces were subcutaneously transplantated into the right back sides of nude mice after anaesthetized by Afferden, randomly. After the tumors reached about 100 mm^3^, mice were divided into 3 groups and received intralesional injection either with DMSO or WB-308 (10 mg/kg/day per mouse and 50 mg/kg/day per mouse) every day for 27 days. During the administration of WB-308, the body weight and the tumor size of the mice were monitored every 3 days. Mice were continually observed until they were sacrificed [45].

### Immunohistochemistry staining

Immunohistochemistry was performed as reported previously [46]. In brief, xenograft tumors in lungs were excised, fixed, and embedded in paraffin. Sections were stained with anti-proliferating marker Ki-67 (Santa Cruz Biotechnology) and Bxl-xL (CST). And the phosphorylation of EGFR and ERK1/2 were also stained. Immunohistochemical staining was performed according to standard protocol. Images were taken with Leica microscope (Leica, DM4000B). The results were analyzed using Image-Pro Plus 6.0 software [47].

### Statistical analysis

Results were statistically analyzed using the Student's *t*-test with Microsoft Excel. All experiments were repeated at least three times. A value of *p* < 0.05 was considered statistically significant.

## SUPPLEMENTARY FIGURE


