# Supplementary material for: Inhibition of non-small cell lung cancer (NSCLC) growth by a novel small molecular inhibitor of EGFR
Source: Oncotarget. 2015 Feb 5;6(9):6749–61. doi: 10.18632/oncotarget.3155 (PMC4466647; doi:10.18632/oncotarget.3155)
Supplement: Supplementary file 1 [file oncotarget-06-6749-s001.pdf]

SUPPLEMENTARY FIGURE

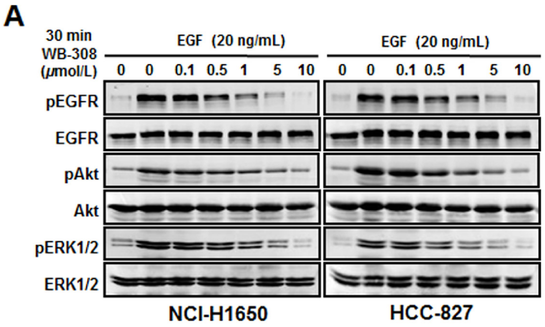

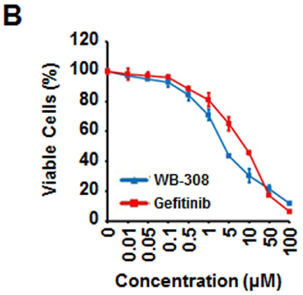

Supplementary Figure 1: (A) WB-308 suppresses the EGFR signaling pathway in NSCLC cells NCI-H1650 and HCC-827. **(B)** WB-308 suppresses the patient-derived spinal metastasized tumor cell proliferation. The patient-derived spinal metastasized tumor cells were separated and subjected to the cell viability assay stained by SRB as described in Materials and Methods. Columns, mean (n = 10); bars, SE (n = 3; t-test, $P < 0.05$).
